# Uniting Gold to Amalgam in Tooth Filling

**Published:** 1883-03

**Authors:** N. W. Kingsley

**Affiliations:** New York.


### ARTICLE VIII.

### *Uniting Gold to Amalgam in Tooth Filling.*

BY N. W. KINGSLEY, D. D. S., OF NEW YORK.

[Reported to the N. Y. Odontological Society.]

I would like to refer to a use I have been making of amalgam for several years, and which I reported to the State Society some four or five years ago. I advocated at that time that in many instances of large cavities on approximal surfaces,—of bicuspids and molars particularly,—they should be partly filled with amalgam, to be followed at the same sitting with gold, carrying out the filling to the grinding-surface of the tooth, if one desired to avoid a full amalgam filling. Without going into a lengthy discussion of the arguments in favor, or the objections that might be offered, let me say that I have seen a great many of those fillings, and have watched them with much solicitude and anxiety, but I have never seen a single instance where there has been any recurrence of decay at the cervical edge of the cavity. In nearly every case that I have seen, I have found the amalgam discolored on the surface, but the gold as bright as any gold filling, and the tooth preserved perfectly from further decay. I have been querying, why this result; whether it was attributable entirely to the fact that with the amalgam I got perhaps a closer adaptation to the cervical edge of the cavity than I would with gold, or whether it was owing to some galvanic condition of the different metals in contact or what? But this I do know, that sooner or later the majority of operations, under like circumstances, when made with gold, are giving out at that portion of the cavity, no matter by whom they are made. I say a majority, not because I have had an opportunity of examining all the fillings made by every operator, not because I am constantly seeing the work of the most skillful operators in gold giving out at the point referred to, within a few years. There is not a dentist who is not seeing this almost daily, and among his own patients, if he would but have the candor to admit it. I have been puzzled for an explanation of the success where amalgam has been used in this manner. There is no evidence of shrinkage of the amalgam that is placed in the upper part of the cavity. Whatever amalgam may do under other circumstances, that amalgam shows no shrinkage after it is put in. It remains perfectly tight, and the tooth does not discolor in contact with it. In filling a tooth in this way, the amalgam occupies a third or a half of the cavity, and the gold is immediately forced into it. For some little time the gold will absorb the mercury and take up all the excess. After that ceases, the gold shows its true gold appearance, and unites or welds in the usual way. The filling can be finished as soon as the gold is packed. The amalgam will be found sufficiently hard to be finished up to the cervical edge. We see here two results: one is that the gold has taken up all the excess of the mercury that can possibly be taken from the mass of amalgam, preventing the possibility of shrinkage; and, secondly, such a filling is in a better condition to make a perfect flush edge at the cervical border. There is little or no risk of the difficulty so often found with gold fillings, of the filling standing out and forming a lodgment for foreign matters, which will end in decay, because the amalgam has not become so hard but that it can be easily brought flush with the body of the tooth. This may be the explanation of the results that I believe will almost invariably attend such operations. I spoke of this before the State Society a few years since with some timidity, for the reason that there is such a prejudice in the minds of almost every one against amalgam,—and especially as the charge is often made that amalgam is only used by those who cannot put in a gold filling. It was not for the reason that gold could not be used, but because I saw gold-fillings were constantly giving out, that I felt obliged to resort to something for the good of the patient, and the result has been so successful and uniform that I felt it a duty to give somebody else the benefit of it.—*Dental Practitioner*.

